# A New Perspective on the Antimicrobial Mechanism of Berberine Hydrochloride Against *Staphylococcus aureus* Revealed by Untargeted Metabolomic Studies

**DOI:** 10.3389/fmicb.2022.917414

**Published:** 2022-07-13

**Authors:** Shu Wu, Kun Yang, Yuhang Hong, Yanju Gong, Jiajia Ni, Ni Yang, Weijun Ding

**Affiliations:** ^1^School of Basic Medical Sciences, Chengdu University of Traditional Chinese Medicine, Chengdu, China; ^2^Key Laboratory of Application of Ecology and Environmental Protection in Plateau Wetland of Sichuan, Xichang University, Xichang, China; ^3^Research and Development Center, Guangdong Meilikang Bio-Sciences Ltd., Dongguan, China; ^4^Dongguan Key Laboratory of Medical Bioactive Molecular Development and Translational Research, Guangdong Medical University, Dongguan, China

**Keywords:** GC-MS untargeted metabolomics, LC-MS untargeted metabolomics, natural antimicrobial, metabolic markers, mechanism

## Abstract

Berberine hydrochloride (BBR) is a natural product widely used in clinical medicine and animal production. It has a variety of antimicrobial effects, but its complex antimicrobial mechanism has not been clarified. This study aimed to discover the metabolic markers and gain a new perspective on the antibacterial mechanism of BBR. The effects of different inhibitory concentrations of BBR on the survival and growth of standard strain *Staphylococcus aureus* ATCC 25923 were analyzed by the bacteriostatic activity test. Differences in intracellular metabolites of *S. aureus* following 19 μg/ml BBR exposure for 1 h were investigated by combining non-targeted metabolomics techniques of gas chromatography-mass spectrometry (GC-MS) and liquid chromatography-mass spectrometry (LC-MS). The results showed that the minimum inhibitory concentration of BBR against *S. aureus* was 51 μg/ml. A total of 368 and 3,454 putative metabolites were identified by GC-MS and LC-MS analyses, respectively. Principal component analysis showed the separation of intracellular metabolite profiles between BBR-exposed samples and non-exposed controls. Pathway activity profiling analysis indicated a global inhibition of metabolisms by BBR exposure, while enhancement was also found in nucleic acid metabolism, amino sugar, and nucleotide sugar metabolism. Several metabolic markers were screened out mainly based on their variable importance of projection values. Two pyridine dicarboxylic acids were significantly downregulated, suggesting the reduction of stress resistance. The oxidized phospholipid (PHOOA-PE) was accumulated, while lipid antioxidant gamma-tocopherol was decreased, and farnesyl PP, the synthetic precursor of another antioxidant (staphyloxanthin), was decreased below the detection threshold. This evidence indicates that BBR reduced the antioxidant capacity of *S. aureus*. Accumulation of the precursors (UDP-GlcNAc, CDP-ribitol, and CDP-glycerol) and downregulation of the key metabolite D-Ala-D-Ala suggest the inhibition of cell wall synthesis, especially the peptidoglycan synthesis. Metabolites involved in the shikimate pathway (such as 3-dehydroshikimate) and downstream aromatic amino acid synthesis were disturbed. This study provides the first metabolomics information on the antibacterial mechanism of BBR against *S. aureus*. The key metabolic markers screened in this study suggest that the shikimate pathway, staphyloxanthin synthesis, and peptidoglycan biosynthesis are new directions for further study of BBR antibacterial mechanism in the future.

## Introduction

*Staphylococcus aureus* is a common bacterial colonizer of human and a variety of animal species which can cause many infectious diseases, such as mastitis, endocarditis, septic arthritis, and sepsis. In the treatment of infectious diseases, antibiotics were initially highly effective, but they currently show several limitations, including widespread resistance, toxicity, and residues. *S. aureus* has developed resistance to various antibiotics including vancomycin (Ragunathan et al., 2018). We need to understand the resistance mechanism of microorganisms to existing antibiotics and develop antibiotics with new antimicrobial mechanisms (Anitha et al., 2016). As complementary or alternative drugs for the treatment or prevention of infection, natural products such as medicinal plants and their extracted components are a good choice (Herrmann et al., 2016; Newman and Cragg, 2016; Kokoska et al., 2019). Some natural compounds are considered to have multiple potential antimicrobial targets (Miryala et al., 2021, 2022). One of the natural anti-infective products is berberine, which is commonly used in clinical medicine, as well as in livestock production and aquaculture.

Berberine is an isoquinoline alkaloid found mainly in the form of hydrochloride (berberine hydrochloride) in many medicinal plants, such as *Coptis chinensis, Phellodendron amurense*, and *Berberis vulgaris* (Imenshahidi and Hosseinzadeh, 2016). It exhibits various pharmacological activities, including prominent anti-inflammatory and anti-infective effects, antagonizing a variety of pathogenic bacteria (including *S. aureus*), fungi, parasites, and even viruses (Shang et al., 2020). Berberine not only can exert an antimicrobial effect by regulating host immunity but also has a significant direct antimicrobial effect. Some experimental evidence shows that berberine alone or in combination with other agents showed obvious antimicrobial effects *in vivo* and *in vitro* (Aksoy et al., 2020; Huang et al., 2020; Xie et al., 2020; Bhatia et al., 2021). Berberine even has the potential to inhibit antibiotic-resistant bacteria (Morita et al., 2016; Zhang et al., 2020; Li et al., 2021), which gives it great potential in the development of new antibiotics. There are many studies on the antimicrobial activity of berberine, and it is found that berberine has a variety of antimicrobial effects. A multi-omics study suggests that berberine may interfere with the nucleic acid, cell wall, cell membrane transport, and motility functions of *Escherichia coli* and inhibit its metabolism (Karaosmanoglu et al., 2014). A recent study has shown that berberine can destroy the cell wall structure and cell membrane integrity of methicillin-resistant *Staphylococcus aureus* (MRSA) and play a synergistic antimicrobial role with clindamycin or rifamycin (Xia et al., 2022). In addition, a few studies have suggested other possible antimicrobial mechanisms of berberine, including the inhibition of biofilm formation, protein synthesis, and bacterial division (Boberek et al., 2010; Li et al., 2011). However, the pathway and potential targets of berberine causing these antibacterial effects are unclear. A more clear direction is needed to further study the complex antimicrobial molecular mechanism of berberine.

Analyzing the expression changes of specific genes in microorganisms is a common method to explain the antimicrobial effect of berberine. The intervention on gene expression level (including transcription and protein synthesis) may be indirect, so it is difficult to explain the antimicrobial mechanism of berberine. Metabolites are the final products of gene expression, and the metabolic alterations represent the exact physiological state of microorganisms (Dorries et al., 2014). Thus, the response of microorganisms to a given antibiotic stress at the metabolic level can help us better understand the antibacterial mechanisms. Several studies have used metabolomics technology to study the antimicrobial mechanism of antibiotics (Dorries et al., 2014; Schelli et al., 2017; Vemula et al., 2017) and natural products (Hussein et al., 2020; Shen et al., 2020; Tang et al., 2021; Chen et al., 2022). However, we have not read any report on the antimicrobial mechanism of berberine based on metabolomics. In this study, berberine hydrochloride (BBR) was used as the representative form of berberine, and we attempted to explore a new perspective of antimicrobial mechanism of berberine by using metabolomics technology. Two untargeted metabolomics techniques, namely, gas chromatography-mass spectrometry (GC-MS) and liquid chromatography-mass spectrometry (LC-MS), were combined to explore the antimicrobial mechanism of BBR against *S. aureus* through extensive screening of metabolic markers and analysis of related metabolic pathways.

## Materials and Methods

### Reagents, Bacterial Strain, and Culture Conditions

The BBR (HPLC ≥ 98%) and vancomycin (potency ≥ 900 μg/mg) were purchased from Desite Bio-Tech Co., Ltd. (Chengdu, CHN) and Solarbio Bio-Tech Co., Ltd. (Beijing, CHN), respectively, and were dissolved in sterilized ultra-pure water before use. The reagents used in the preparation of culture medium were purchased from Aoboxing Bio-Tech Co., Ltd. (Beijing, CHN). The standard strain *S. aureus* ATCC 25923 was purchased from the Biobw Culture Collection (Beijing, CHN). Before use, the strain was subcultured onto nutrient agar plates for 24 h at 37°C. Then, the single colony was selected and coated on a nutritious agar plate to grow for 7 h, and the culture would be used as a “seed” to cultivate working bacteria culture (WBC) for subsequent experiments. For the preparation of WBC, the “seed” was inoculated into the broth medium (composed of 1% tryptone, 0.3% beef extract powder, and 0.5% sodium chloride, pH ~ 7.4) with an initial inoculation dose of 10^7^ CFU/ml and then cultured with shaking at 37°C and 120 rpm for ~2.5 h to enter the logarithmic growth phase (OD_600_ ~ 0.3).

### MIC and MBC Test

The MIC value was determined by the improved broth dilution method to improve the test sensitivity (Surre et al., 2018). A small amount of phenol red (0.018 g/L) and glucose (0.5 %) were added to the broth for the test (containing 1% tryptone, 0.1% beef extract powder, 0.5% sodium chloride, pH ~ 7.4). The basic principle of the test is that bacteria grow to produce acid by fermenting glucose, which makes the broth turn yellow from the original red color. The operation method is similar to that of the traditional broth dilution method except that color change and turbidity change are combined as the basis for judging whether the bacteria grow or not. Generally speaking, the phenol red-containing broth added with a certain concentration of BBR was diluted in a series of gradients and used for the culture of *S. aureus* (~10^8^ CFU/ml) at 37°C. The lowest BBR concentration without visible color change and turbidity change within 24 h was MIC. The bacterial cultures exposed to different concentrations of berberine for 24 h were coated on nutrient agar plates and cultured at 37°C for 24 h. The MBC was determined by the lowest berberine concentration without bacterial growth.

### Antibacterial Effect Test

The WBC (OD_600_ ~ 0.3) was diluted with the same volume of broth medium containing BBR to give an approximate starting inoculum of 10^8^ CFU/ml and to obtain final BBR concentrations of 1/4 × MIC, 3/8 × MIC, 1/2 × MIC, or MIC. The control culture was added with the same volume of broth medium without BBR. The cultures were incubated further for 8 h, and cell growth was monitored spectrophotometrically (OD_600nm_ at 1 h intervals). Time kill tests of the above BBR concentrations were assessed by counting CFU (Lobritz et al., 2015). WBC was incubated in different concentrations of BBR at 37°C for 30 min. Bacterial cells were collected by centrifugation and washed two times with 0.85% sterile saline and then stained for 15 min using Live/Dead BacLight bacterial viability kit (Invitrogen, Eugene, OR, USA) according to the manufacturer's instructions and imaged by Image Xpress Micro Confocal (Molecular Devices LLC, Sunnyvale, CA).

### Cultivation of Metabolic Samples

The BBR concentration (3/8 × MIC, 19 μg/ml) and exposure time (1 h) for the treatment of metabolic samples were determined by the result of time kill tests. The culture conditions were the same as the antibacterial effect test. Therefore, metabolic samples (group T1) were collected at the 1-h time point after being exposed to 19 μg/ml BBR. Two control groups (without BBR addition) collected at zero time point were treated as the initial control group (C0) and those collected 1 h later were treated as the growth control group (C1).

### Intracellular Metabolites Extraction

Intracellular metabolites were extracted using cold methanol and chloroform (Stipetic et al., 2016). Briefly, after co-incubation with or without BBR, 80 ml of bacterial culture from each biological sample (six biological replicates for each group) was collected. The bacterial cells were harvested by 4°C cryogenic centrifugation at 6,000 rpm for 10 min, followed by cold phosphate buffer saline (PBS) washes. A total of 1 ml cold methanol:water (4:1, v/v) and 200 μl of chloroform were added to the cell pellet, and the mixtures were vortexed. After that, cells were broken up with an ultrasonic homogenizer (3 min, 500 W), and 20 μl of L-2-chlorophenylalanine (0.3 mg/ml) was added as the internal standard. Then, the mixture was extracted by ultrasonic for 20 min in ice-water bath and then centrifuged at 4°C (13,000 rpm) for 10 min. Finally, 200 μl of the supernatant for GC-MS (or 400 μl for LC-MS) was dried in a freeze concentration centrifugal dryer. Quality control sample (QC) was prepared by mixing an aliquot of all samples to be a pooled sample.

### Untargeted Metabolomics Analyses

A Thermo Trace 1310/TSQ 9000 GC/MSD System was used for GC-MS analysis. DB-5MS fused-silica capillary column (30 m × 0.25 mm × 0.25 μm) was utilized to separate the derivative metabolites. For LC-MS analysis, the freeze-dried samples were re-extracted and analyzed by a Nexera UPLC system coupled with Q Exactive quadrupole-orbitrap mass spectrometer equipped with heated electrospray ionization (ESI) source. An ACQUITY UPLC HSS T3 column (1.8 μm, 2.1 × 100 mm) was employed in both positive and negative modes. Metabolome analysis was performed by Lu-Ming Biotech Co. Ltd. (Shanghai, China) [see details in the supporting information (Supplementary Text 1)].

### Metabolome Data Processing

The MS-DIAL software was used to preprocess GC-MS data. Metabolite characterization is based on the LUG database (a self-built untarget database of GC-MS from Lu-Ming Bio). All peak signal intensities in each sample were segmented and normalized according to the internal standards with RSD > 0.3 after screening. LC-MS data were processed by software Progenesis QI V2.3. Main parameters of 5 ppm precursor tolerance, 10 ppm product tolerance, and 5% production threshold were applied. Compound identification was based on the precise mass-to-charge ratio (m/z), secondary fragments, and isotopic distribution using HMDB, Lipidmaps (V2.3), Metlin, EMDB, PMDB, and self-built databases to do qualitative analysis. Peaks with a missing value (ion intensity = 0) in more than 50% of groups were removed. Zero value was replaced by half of the minimum value. Compounds with resulting scores below 36 (out of 60) points were deemed to be inaccurate and removed.

### Statistical Analyses

The data matrix of the metabolome was imported in R to carry out principle component analysis (PCA) and orthogonal partial least-squares-discriminant analysis (OPLS-DA). To prevent overfitting, 7-fold cross-validation and 200 response permutation testing (RPT) were carried out to evaluate the quality of the OPLS-DA models. Variable importance of projection (VIP) values obtained from the OPLS-DA model were used to rank the overall contribution of each variable to group discrimination. A two-tailed Student's *T*-test was further used to verify whether the metabolites of difference between the two groups were significant. *P*-value adjustment was performed using the Benjamini-Hochberg method (false discovery rate). Heatmaps (hierarchical clustering) were plotted by http://www.bioinformatics.com.cn, a free online platform for data analysis and visualization. Metabolomics datasets and metabolic pathway activities were correlated using the pathway activity profiling (PAPi) algorithm (Aggio, 2014). Only the significant metabolites (VIP > 1, FC > 2, *P* < 0.05 and adj. *P* < 0.05) were analyzed by the PAPi_1.8.0 package in R.

## Results

### Activity of BBR Against *S. aureus*

The MIC value of BBR against *S. aureus* strain ATCC 25923 was 51 μg/ml, and the MBC value was two times the value of MIC (102 μg/ml). When combined with vancomycin, BBR of 1/2 × MIC (25.5 μg/ml) halved the MIC value (2 μg/ml) of vancomycin against *S. aureus* strain ATCC 25923 (Supplementary Figure 1). Fluorescent staining of live and dead bacterial cells showed that BBR had a significant inhibitory effect on the survival of *S. aureus* (Supplementary Figure 2). BBR inhibited the growth of *S. aureus* in a concentration-dependent manner. No obvious growth trend was observed in *S. aureus* exposed to MIC and 1/2 × MIC of BBR, while moderate inhibition and mild inhibition were mediated by 3/8 × MIC and 1/4 × MIC, respectively (Supplementary Figure 3A). Based on the results of time-kill test (Supplementary Figure 3B), BBR concentration and exposure time for the treatment of metabolic samples were determined. The exposure time of 1 h was chosen because BBR had shown obvious bactericidal activity at this time (Supplementary Figure 3B); 3/8 × MIC (19 μg/ml) was a suitable exposure concentration to minimize cell death at the 1-h time point and to achieve an obvious bacteriostatic effect at later time points (Supplementary Figure 3B).

### Metabolite Profiles

**Figure 1:**
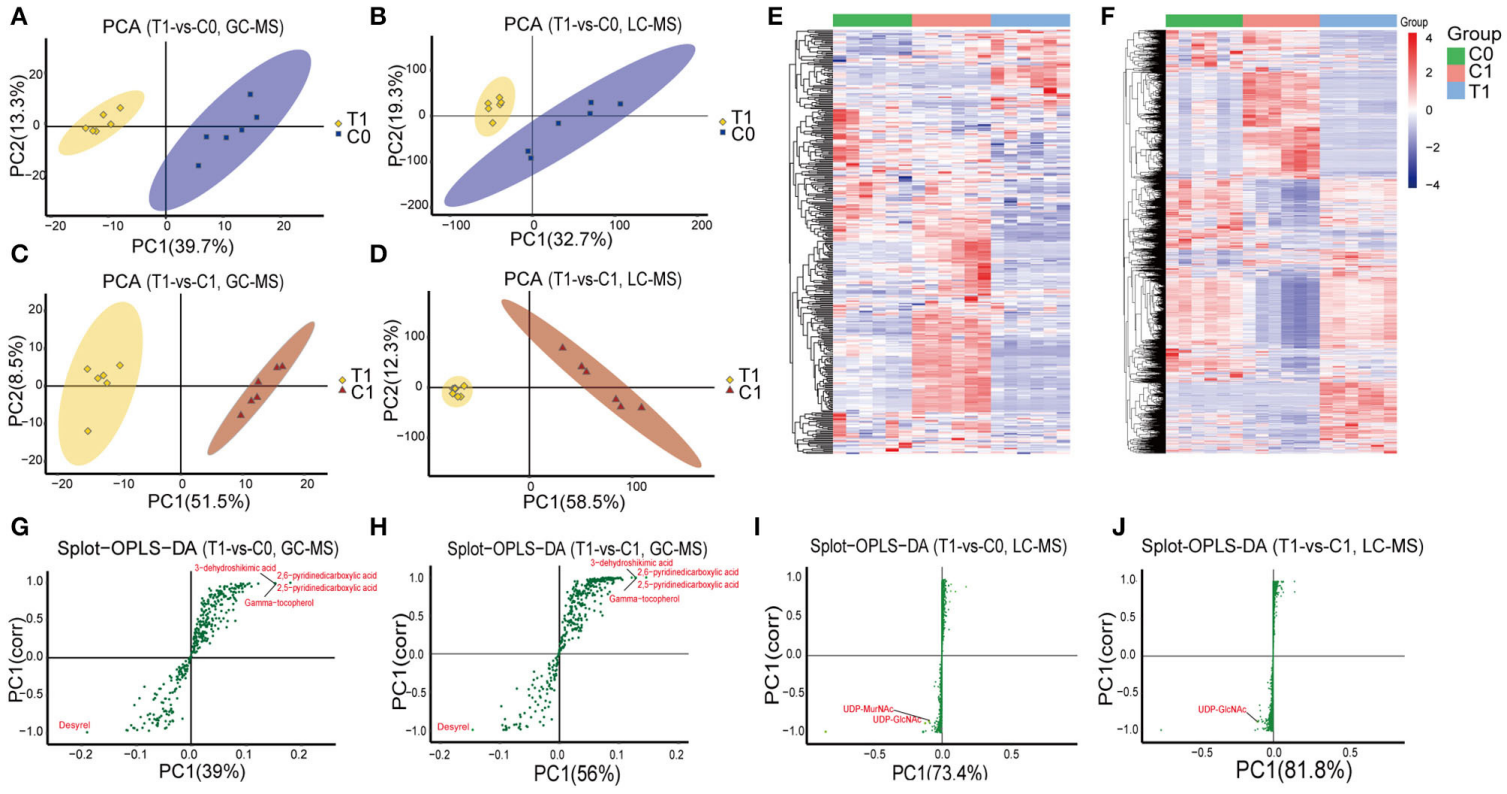
Overview of metabolomics analysis. **(A–D)** PCA analysis; **(E, F)** Heatmaps of identified metabolites; **(G–J)** Graphs of OPLS-DA model, points farther from the origin represent metabolites that contribute more to group discrimination. T1: BBR-exposed group; C0: initial control group; C1: growth control group.

A total of 368 and 3,454 putative metabolites were identified, respectively, from GC-MS and LC-MS datasets. They were divided into 16 super classes including 2,296 classified metabolites and 1,526 unclassified metabolites (Table 1). PCA reviewed a significant separation between controls [initial control (C0) and growth control (C1)] and BBR-exposed group (T1) (Figures 1A–D). A pair of heatmaps demonstrated the characteristic profiles of T1 group (Figures 1E,F). OPLS-DA models showed the importance of each metabolite on group discrimination (Figures 1G–J). Model parameters indicated the excellent prediction ability of the models (Supplementary Table 1), and no overfitting was found (Supplementary Table 1, Supplementary Figure 4).

**Table 1:** Classification statistics of identified metabolites.

**Super Class of metabolites**	**GC-MS**	**LC-MS**	**Total**
Lipids and lipid-like molecules	62	637	698
Organic acids and derivatives	76	384	460
Organoheterocyclic compounds	38	265	303
Organic oxygen compounds	56	227	283
Benzenoids	31	195	226
Phenylpropanoids and polyketides	13	111	124
Nucleosides, nucleotides, and analogs	15	77	92
Organic nitrogen compounds	12	30	42
Organosulfur compounds	0	25	25
Hydrocarbons	1	17	18
Alkaloids and derivatives	1	11	12
Homogeneous non-metal compounds	3	2	5
Organohalogen compounds	0	4	4
Lignans, neolignans and related compounds	0	2	2
Organic 1,3-dipolar compounds	0	1	1
Organometallic compounds	0	1	1
Unclassified	61	1,465	1,526

### Key Metabolic Pathways Affected by BBR

**Figure 2:**
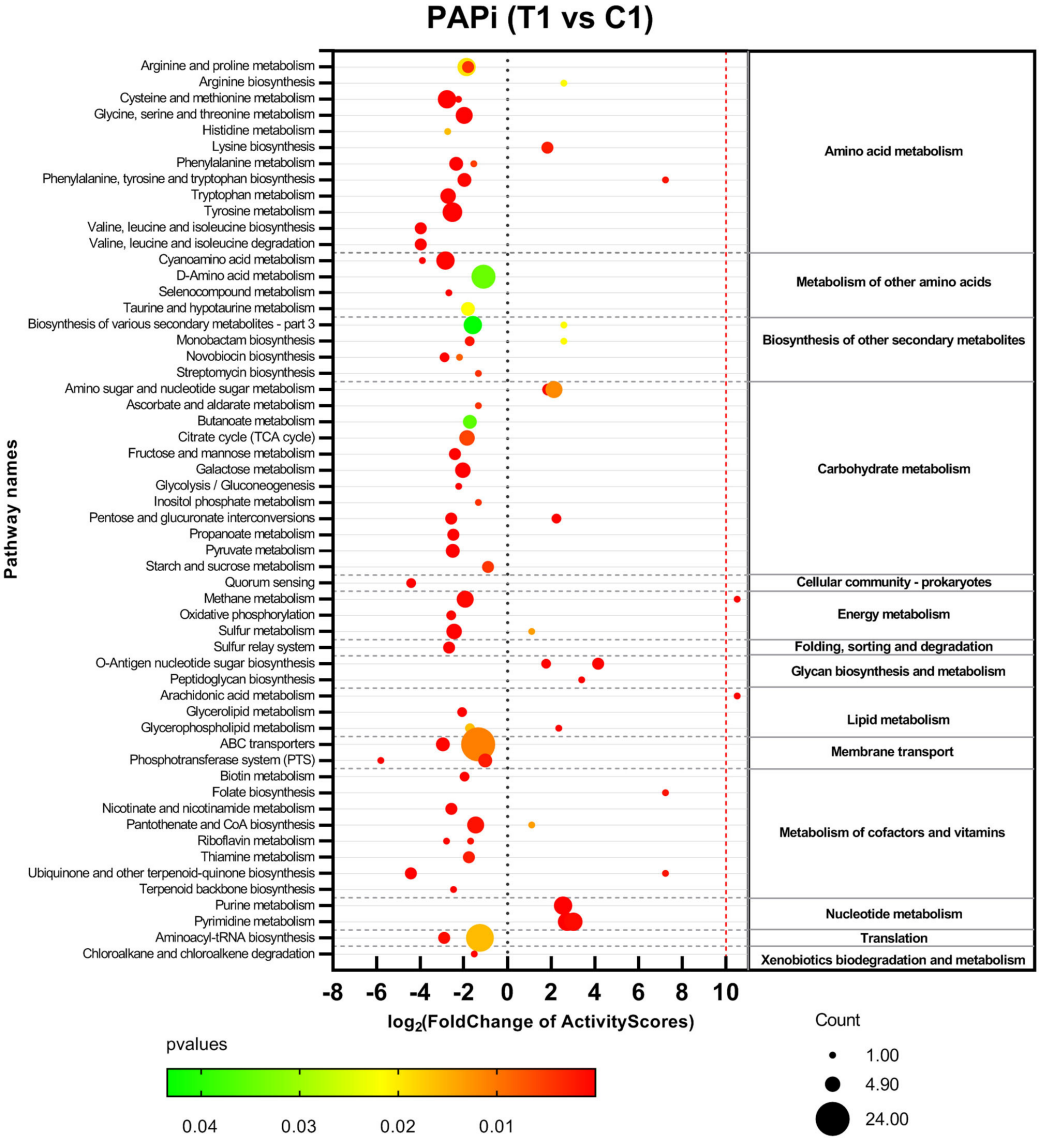
Activities profile of metabolic pathways in comparison with BBR-exposed group (T1) vs. growth control group (C1). Results from GC-MS and LC-MS datasets were merged. The color and size of bubbles represent the statistical significance and the number of metabolite species matched to the pathways, respectively.

**Figure 3:**
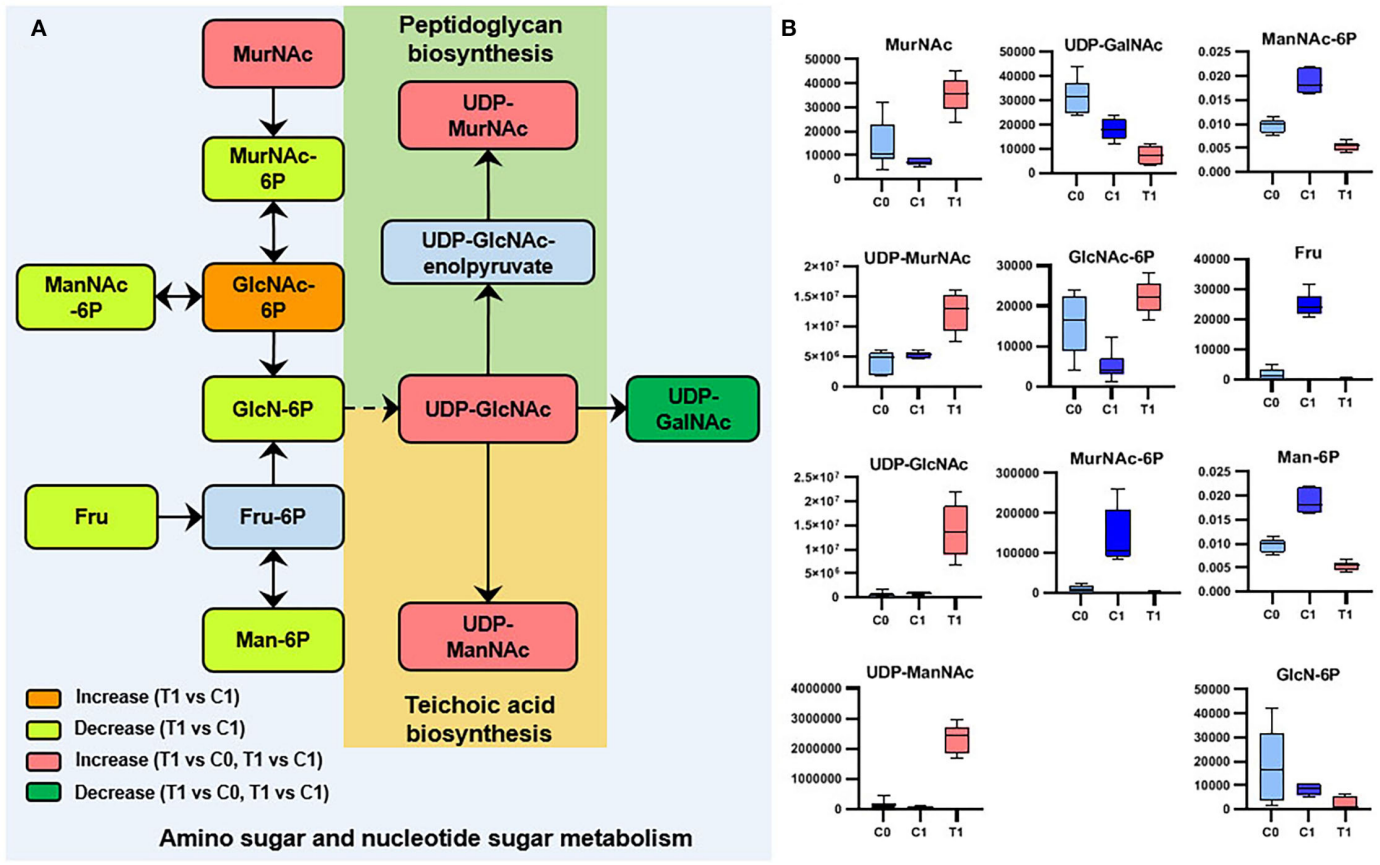
Significant metabolites involved in amino-sugar and sugar-nucleotide metabolism. **(A)** Relationship among metabolites. T1: BBR-exposed group; C0: initial control group; C1: growth control group. **(B)** Box plots for metabolites marked in A (|log_2_FC| > 1; *P* < 0.05; adj. *P*-value <0.05).

Metabolomics datasets and metabolic pathway activities were correlated using PAPi analysis. As compared with control groups, the activities of most metabolic pathways in the T1 group were downregulated (Figure 2, Supplementary Figure 5). The upregulated metabolic pathways were mainly nucleic acid metabolism, amino sugar, and nucleotide sugar metabolism, as well as glycan biosynthesis and metabolism. Similar trends can be seen in the volcanic maps (Supplementary Figure 6). The enhancement of nucleic acid metabolism activity involved the accumulation of nucleotide metabolites (Supplementary Figure 6). The upregulation of amino sugars, nucleotide sugars metabolism, and downstream glycan biosynthesis involved the accumulation of precursor metabolites of glycan biosynthesis, mainly UDP-N-acetylmuraminate (UDP-MurNAc), UDP-N-acetylglucosamine (UDP-GlcNAc), and UDP-N-acetyl-D-mannosamine (UDP-ManNAc) (Figure 3).

### The Metabolites of Cell Wall Biosynthesis Were Disturbed

**Figure 4:**
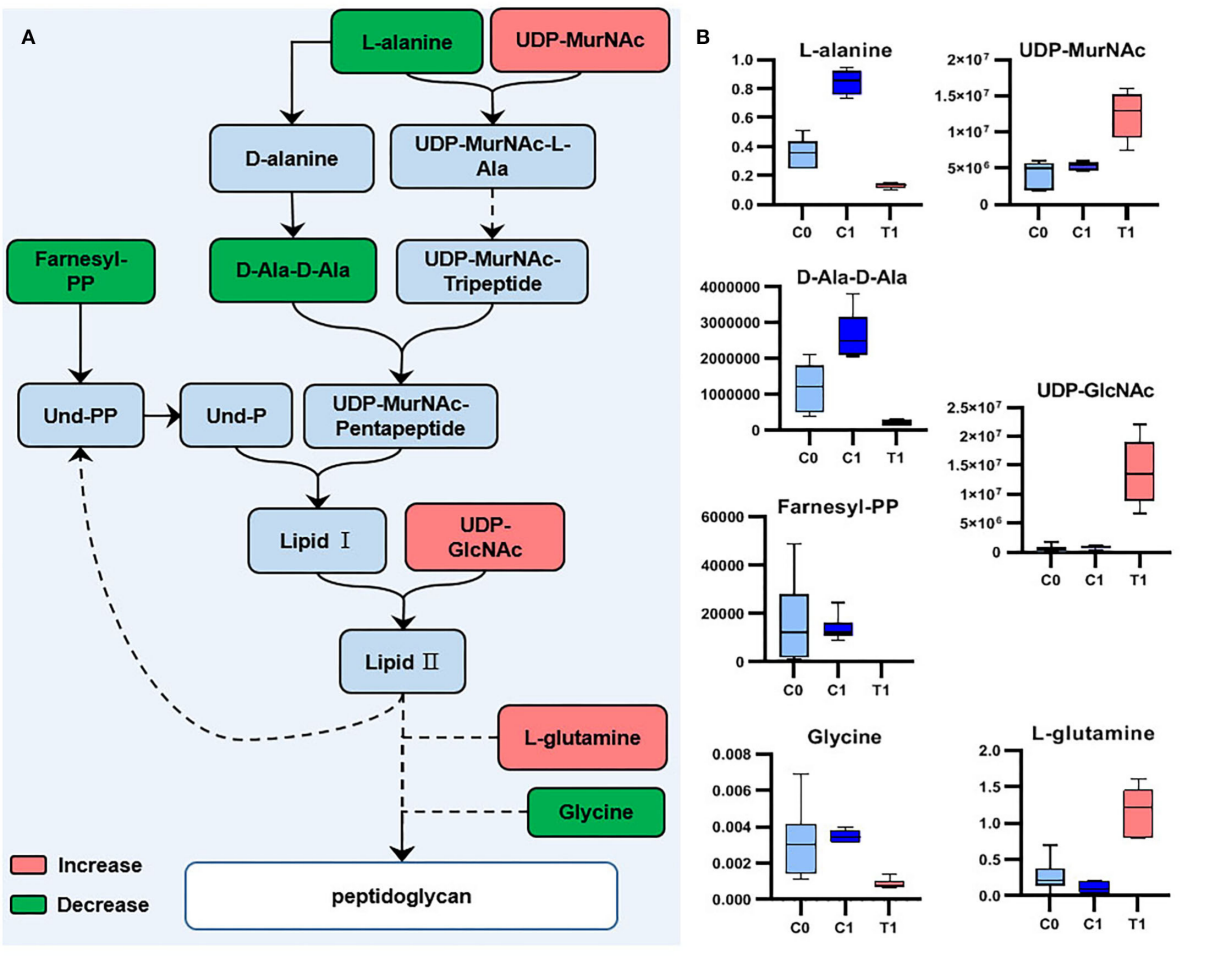
Significant metabolites involved in peptidoglycan biosynthesis. **(A)** Relationship among metabolites. **(B)** Box plots for significant metabolites (|log_2_FC| > 1; *P* < 0.05; adj. *P*-value <0.05).

We analyzed the potential biological functions of the top five metabolites sorted by VIP values and the related metabolites (Table 2, Supplementary Figure 7). UDP-GlcNAc (a precursor of peptidoglycan and teichoic acid) was one of the VIP-top five metabolites in the LC-MS dataset and was significantly upregulated in the T1 group [log_2_FC = 4.6 (4.0)]. Six other significant metabolites involved in peptidoglycan biosynthesis were identified (Table 2), and their relationships are shown in Figure 4. Downregulated D-alanyl-D-alanine (D-Ala-D-Ala) [log_2_FC = −2.4 (−3.6)] restricts the upstream part of peptidoglycan biosynthesis pathway. Farnesyl diphosphate (farnesyl-PP), the precursor metabolite of undecaprenyl pyrophosphate (Und-PP) in the peptidoglycan biosynthesis pathway, was found to be decreased below the detection threshold in the T1 group, while it was detected in all the 12 samples of the control groups [log_2_FC = −29.5 (−29.3)]. L-alanine [log_2_FC = −1.4 (−2.7)] and glycine [log_2_FC = −1.9 (−2.0)] were downregulated, while L-glutamine was accumulated [log_2_FC = 2.1 (3.4)]. The precursors of teichoic acid biosynthesis (UDP-GlcNAc, UDP-ManNAc, CDP-ribitol, and CDP-glycerol) were also accumulated significantly (Table 2, Supplementary Figure 7).

**Table 2:** The main molecular markers in BBR-exposed *S. aureus*.

**Metabolites**	**Database**	**Classification**	**VIP [ T1-vs.-C0 (C1)]**	**Log_**2**_FC [T1-vs.-C0 (C1)]**	**Main Pathways/Functions**
Desyrel	GC-MS	Phenylpiperazines	3.6 (2.8)	8.9^#^ (8.9)*	unknown
2,5-pyridinedicarboxylic acid	GC-MS	Pyridinecarboxylic acids	3.5 (2.8)	−8.3^#^ (−9.0)*	Stress resistance
2,6-pyridinedicarboxylic acid	GC-MS	Pyridinecarboxylic acids	3.5 (2.8)	−8.3^#^ (−9.0)*	Stress resistance
Gamma-tocopherol	GC-MS	Tocopherols	3.0 (2.5)	−6.0^#^ (−6.9)*	Lipid antioxidant
3-dehydroshikimate	GC-MS	Cyclohexenones	3.0 (2.5)	−5.9^#^ (−6.9)*	Shikimate pathway
2′,2′-Dimethyl(pyrano-5′,6′:3:4)-1,5-dihydroxy-6-methoxy-10-methylacridone	LC-MS	Acridones	83.0 (75.7)	12.3^#^ (14.1)*	Unknown
UDP-GlcNAc	LC-MS	Pyrimidine nucleotide sugars	11.9 (10.7)	4.6^#^ (4.0)*	Peptidoglycan and teichoic acid biosynthesis
Gravacridonediol methyl ether	LC-MS	Acridones	11.4 (10.4)	14.6^#^ (16.3)*	Unknown
UDP-MurNAc	LC-MS	Pyrimidine nucleotide sugars	9.1 (7.7)	1.5^#^ (1.2)*	Peptidoglycan and teichoic acid biosynthesis
CDP-ribitol	LC-MS	Nucleotide-alditol	6.7 (6.8)	1.5^#^ (2.2)*	Teichoic acid biosynthesis
UDP-ManNAc	LC-MS	Pyrimidine nucleotide sugars	5.0 (4.7)	4.0^#^ (5.1)*	Teichoic acid biosynthesis
CDP-glycerol	LC-MS	Glycerophospholipids	4.2 (3.9)	2.4^#^ (2.3)*	Teichoic acid biosynthesis
D-Ala-D-Ala	LC-MS	Dipeptides	3.0 (4.8)	−2.4^#^ (−3.6)*	Peptidoglycan biosynthesis
L-Glutamine	GC-MS	Amino acids	1.7 (1.7)	2.1^#^ (3.4)*	Peptidoglycan biosynthesis
Glycine	GC-MS	Amino acids	1.4 (1.3)	−1.9^#^ (−2.0)*	Peptidoglycan biosynthesis
L-Alanine	GC-MS	Amino acids	1.4 (1.5)	−1.4^#^ (−2.7)*	Peptidoglycan biosynthesis
Chorismate	LC-MS	Dicarboxylic acids and derivatives	1.2 (1.1)	6.3^#^ (7.2)*	Shikimate pathway/Phenylalanine, tyrosine and tryptophan biosynthesis/Folate biosynthesis
PHOOA-PE	LC-MS	Oxidized glycerophospholipids	1.2 (1.1)	32.5^#^ (32.5)*	Marker of lipid peroxidation
Farnesyl-PP	LC-MS	Sesquiterpenoids	0.3 (0.3)	−29.5 (−29.3)*	Staphyloxanthin and peptidoglycan biosynthesis

^*^Statistically significant (P <0.05, adj. P-value <0.05) for berberine-exposed (T1) group compared with initial control (C0) group.

*^#^Statistically significant (P <0.05, adj. P-value <0.05) for T1 group compared with growth control (C1) group*.

### The Metabolites Related to Stress Resistance and Antioxidant Activity Were Significantly Downregulated

The VIP-top five metabolites in GC-MS dataset were desyrel, 2,5-pyridinedicarboxylic acid, 2,6-pyridinedicarboxylic acid, gamma-tocopherol, and 3-dehydroshikimate (Table 2, Supplementary Figure 7). 2,5-pyridinedicarboxylic acid [log_2_FC = −8.3 (−9.0)] and 2,6-pyridinedicarboxylic acid [log_2_FC = −8.3 (−9.0)] were significantly decreased after BBR treatment. They belong to pyridine dicarboxylic acid, which is known to be abundant in bacterial spores and endows spores with stress resistance (Setlow, 2007; Ramirez-Guadiana et al., 2017). Gamma-tocopherol was downregulated in the T1 group [log_2_FC = −6.0 (−6.9)], which is known as a lipid vitamin and acts as an antioxidant (Kim et al., 2012). The decreased farnesyl-PP in the T1 group, as the precursor of Und-PP, is also the precursor of staphyloxanthin (another important antioxidant in *S. aureus*). The oxidized glycerol phospholipid (PHOOA-PE) was not detected in the control groups, but was accumulated in the T1 group (log_2_FC = 32.5).

### The Metabolites Involved in the Shikimate Pathway and Biosynthesis of Aromatic Amino Acids Were Disturbed

**Figure 5:**
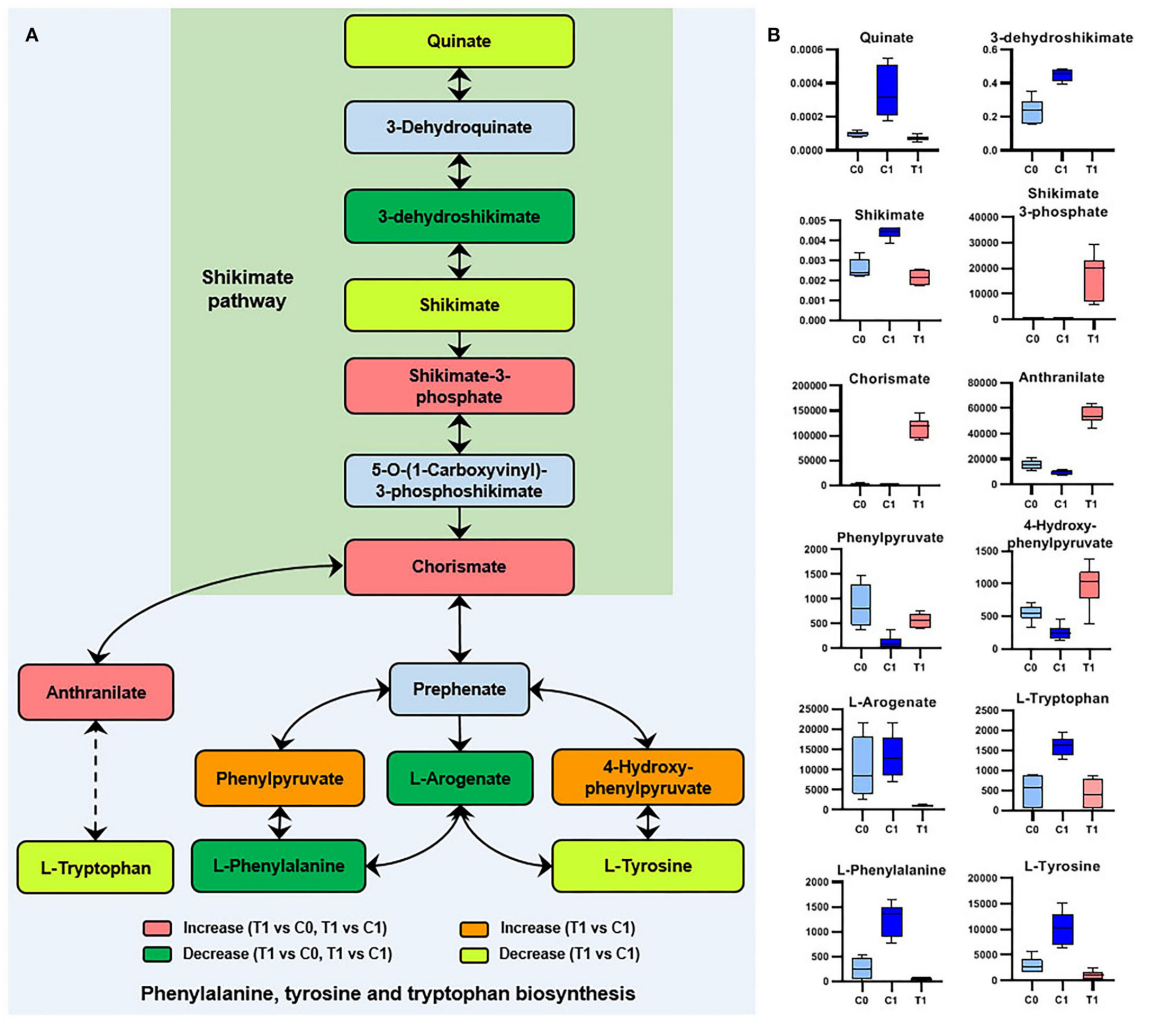
Significant metabolites involved in phenylalanine, tyrosine, and tryptophan biosynthesis. **(A)** Relationship among metabolites. T1: BBR-exposed group; C0: initial control group; C1: growth control group. **(B)** Box plots for metabolites marked in A (|log2FC| > 1; *P* < 0.05; adj. *P*-value <0.05).

The two metabolites involved in the shikimate pathway, 3-dehydroshikimate [log_2_FC = −5.9 (−6.9)] and chorismate [log_2_FC = 6.3 (7.2)], showed opposite regulatory trends. L-phenylalanine, L-tyrosine, and L-tryptophan, which were synthesized using chorismate as precursors, were downregulated (Figure 5).

## Discussion

According to the existing research, the antibacterial effect of berberine can be generally summarized as “multi-effect” (Karaosmanoglu et al., 2014). Berberine can inhibit the formation of biofilm (Zhang et al., 2020; Bhatia et al., 2021; Xu et al., 2021), destroy the cell wall and cell membrane (Karaosmanoglu et al., 2014; Zhang et al., 2020; Xia et al., 2022), inhibit bacterial cell division (Domadia et al., 2008), inhibit nucleic acid and protein synthesis (Du et al., 2020), and inhibit bacterial adhesion (Kang et al., 2015), etc.

This study revealed that BBR inhibited most of the metabolic activities of amino acid metabolism, carbon metabolism, and energy metabolism in *S. aureus*, while the accumulation of nucleotide metabolites suggested that the synthesis of downstream nucleic acid macromolecules may be inhibited (Figure 2). The metabolic changes in these pathways support the inhibitory effect of berberine on bacterial growth proposed by previous studies. However, these metabolic changes are not the unique antimicrobial properties of berberine, as exposure to other antibiotics also causes a general perturbation of bacterial metabolism (Dorries et al., 2014). Berberine and its derivatives inhibit bacterial division by inhibiting the cell division protein FtsZ (Boberek et al., 2010; Sun et al., 2014). Although the results of this study cannot directly verify the target of BBR inhibiting bacterial cell division, the extensive changes in bacterial metabolism caused by BBR may be the result of complex physiological regulation indirectly caused by the inhibition of bacterial cell division and other possible antibacterial activities of BBR.

The main metabolic markers screened in this study provide a new perspective for exploring the antibacterial mechanism of BBR, as follows.

### Shikimate Pathway

The end product of the shikimate pathway (chorismate) was accumulated (Figure 5), indicating a disturbance in the biosynthesis of downstream aromatic amino acids and folate. In contrast, the intermediate metabolite (3-dehydroshikimate) of the shikimate pathway was significantly downregulated by BBR (Figure 5). Since being essential in bacteria and fungi, but absent from mammals, the shikimate pathway was suggested to be an antimicrobial target (Sadaka et al., 2018). Therefore, it is worthwhile to further study the interference mechanism of BBR on the bacterial shikimate pathway.

### Antioxidant Capacity

**Figure 6:**
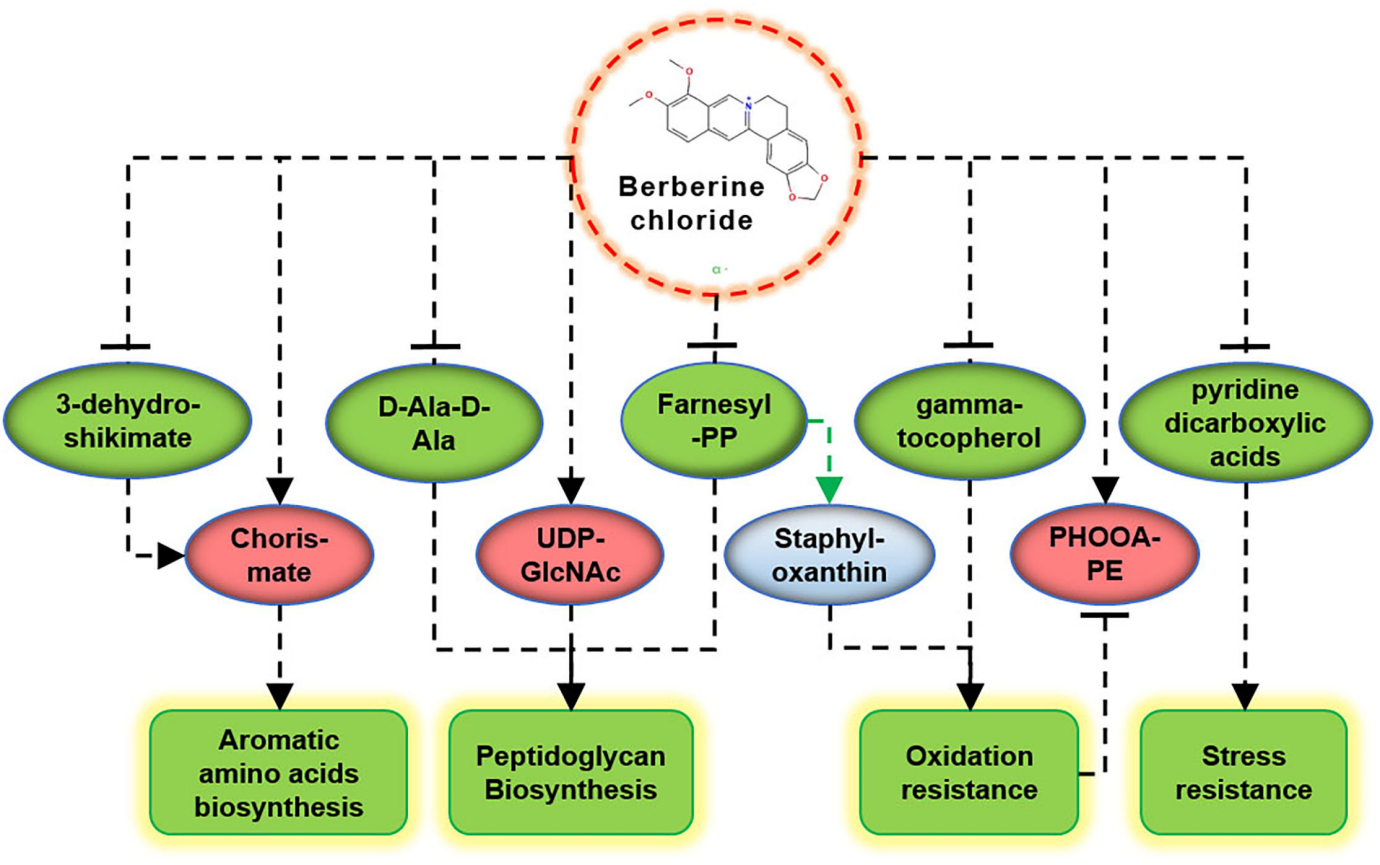
Schematic diagram of the key metabolites in *S. aureus* in response to BBR exposure. Upregulation (promoting) and downregulation (inhibiting) are shown in red and green colors, respectively.

Bactericidal antibiotics cause bacterial death due to oxidative damage by stimulating the generation of reactive oxygen species (ROS) (Kohanski et al., 2007), which is also applicable to the action mode of BBR (Du et al., 2020). In this study, some evidence suggests that BBR reduces the antioxidant capacity of *S. aureus*, such as the reduction of gamma-tocopherol and farnesyl-PP, and the accumulation of PHOOA-PE (Figure 6). Gamma-tocopherol reacts with ROS and protects unsaturated fatty acids from oxidation (Kim et al., 2012). Farnesyl-PP is a precursor metabolite of staphyloxanthin that acts as an antioxidant to protect *S. aureus* from being killed by ROS released by leukocytes (Clauditz et al., 2006). The downregulation of gamma-tocopherol and farnesyl-PP means the decrease in antioxidant capacity, and as a result, the oxidized glycerophospholipid (PHOOA-PE) was accumulated in the cells of *S. aureus*. Staphyloxanthin is the main pigment in *S. aureus*, and its accumulation is inhibited by BBR (Supplementary Figure 8). In addition, based on the transcriptome data of our related study (unpublished), the transcription levels of genes involved in staphyloxanthin biosynthesis were also downregulated by BBR (Supplementary Figure 9). The targeted perturbation of staphyloxanthin is expected to restore the sensitivity of drug-resistant strains to traditional antibiotics (Garcia-Fernandez et al., 2017; Hui et al., 2020). Therefore, the attractive potential of BBR to inhibit staphyloxanthin synthesis is another direction worthy of further study.

The synthesis of farnesyl-PP is regulated by other upstream metabolic pathways (Lan et al., 2010). Since BBR interferes with most of the metabolic activities of *S. aureus*, its effect on the synthesis of farnesyl-PP is complex. As a key precursor metabolite that likes the biosynthesis of staphyloxanthin and peptidoglycan, farnesyl PP affects the abilities of antioxidation and cell wall synthesis, both of which are critical to the survival of *S. aureus*.

### Cell Wall Synthesis

A recent study found that berberine can damage the cell walls of MRSA, but the mechanism is unknown (Xia et al., 2022). According to the metabolic responses of *S. aureus* to antibiotics with known targets, the precursor metabolites upstream would accumulate, while the level of metabolites downstream of the inhibited metabolic targets would decrease (Schelli et al., 2017; Vemula et al., 2017). In this study, besides farnesyl PP, the change of other metabolites also demonstrated that BBR act by impairing bacterial cell wall synthesis. The accumulation of precursor metabolites related to cell wall synthesis, such as UDP-GlcNAc, CDP-ribitol and CDP-glycerol, indicated that the downstream synthesis pathways of peptidoglycan and teichoic acid were inhibited. The level of D-Ala-D-Ala was significantly decreased, suggesting the inhibition of BBR to the early stage of peptidoglycan biosynthesis. The synthetic pathway of D-Ala-D-Ala is one of the targets of the cell wall-targeting antibiotics, such as D-cycloserine and D-boroalanine, which act through the inhibition of D-Ala-D-Ala-ligase (Vemula et al., 2017). Whether BBR has the same target as these two antibiotics needs further verification. Since BBR has synergistic antibacterial effects with other antibiotics such as clindamycin, rifamycin (Xia et al., 2022), and ampicillin (Dziedzic et al., 2015), and we confirmed the synergistic antibacterial effect of BBR combined with vancomycin (which also targets the synthesis of peptidoglycan) (Supplementary Figure 1), its inhibitory effect on the synthesis of peptidoglycan is an interesting discovery and has important value for further study.

### Prospects

Some of the metabolites identified in this study, such as the pyridine dicarboxylic acids, have unclear metabolic pathways in *S. aureus*. Pyridine dicarboxylic acid is present in the spores of spore-producing bacteria in high concentrations and endorses the spores with stress-resistant properties (Setlow, 2007). The genes of dihydro-pyridine dicarboxylate synthases and reductase had been annotated in the genome of MRSA, and they were considered to have potential interactions with the penicillin-resistant binding protein (PBP2a) (Yuan et al., 2010). However, the properties of the substrates of these enzymes remain controversial (Devenish et al., 2010; Karsten et al., 2018). It is worth mentioning that gamma-tocopherol can be synthesized in plants and algae with farnesyl-PP as a precursor (56), although no study on its biosynthesis in *S. aureus* has been retrieved. It is predicted that the average number of metabolites of *S. aureus* ranges from more than 1,000 to more than 2,000, with a maximum of 4,416 (Renz and Drager, 2021). Among the more than 3,000 metabolites identified in this study, a considerable part of them should be products of unknown metabolic pathways, in addition to substances absorbed from the culture environment. As metabolic databases become richer, the number of metabolites accurately identified will increase. Therefore, the revelation of unknown metabolic pathways in the future will help us to deeply understand the complexity and intrinsic relationship of antibiotic effects on bacterial metabolism. In addition, when there is a clear research direction or determined metabolic pathway (as suggested in this study), the application of targeted metabolomics and other technologies that can accurately verify the specific antibacterial effect will further promote the research on the antibacterial mechanism of natural products such as BBR.

## Conclusion

The key metabolic markers suggest that BBR plays antibacterial activities against *S. aureus* ATCC 25923 by inhibiting the cell wall biosynthesis, promoting oxidative damage, reducing stress resistance, and inhibiting the synthesis of aromatic amino acids (Figure 6). These results provide new directions of peptidoglycan biosynthesis, staphyloxanthin synthesis, and shikimate pathway to study the antibacterial molecular mechanism of BBR and help to promote its application in the field of anti-infective medicine.

## Data Availability Statement

The original contributions presented in the study are included in the article/Supplementary Material, further inquiries can be directed to the corresponding author/s.

## Author Contributions

SW, KY, and NY conducted the experiments. SW, YH, and JN performed data analysis. SW and YG wrote the manuscript. SW and WD reviewed, edited the manuscript, and acquired funding. All authors made significant contributions to this article and have read and agreed to the final manuscript.

## Funding

This study was supported by the Sichuan Science and Technology Program (No. 2021YJ0112) and the Chengdu University of TCM Science Foundation (BSH2019010). The authors declare that this study received funding from Science and Technology Department of Sichuan Province and Chengdu University of TCM. The funders were not involved in the study design, collection, analysis, interpretation of data, the writing of this article or the decision to submit it for publication.

## Conflict of Interest

JN was employed by Guangdong Meilikang Bio-Sciences Ltd. The remaining authors declare that the research was conducted in the absence of any commercial or financial relationships that could be construed as a potential conflict of interest.

## Publisher's Note

All claims expressed in this article are solely those of the authors and do not necessarily represent those of their affiliated organizations, or those of the publisher, the editors and the reviewers. Any product that may be evaluated in this article, or claim that may be made by its manufacturer, is not guaranteed or endorsed by the publisher.

## Supplementary Material

Supplementary Text 1 Details for untargeted metabolomics analyses.

Supplementary Figure 1 MIC test of BBR combined with vancomycin against *S. aureus* ATCC 25923.

Supplementary Figure 2 The effect of BBR on survival of *S. aureus* ATCC 25923.

Supplementary Figure 3 Growth curves **(A)** and the results of time kill tests **(B)** for *S. aureus* ATCC 25923 in the presence (1/4MIC, 3/8MIC, 1/2MIC or MIC) or absence (control) of berberine.

Supplementary Figure 4 Result of RPT.

Supplementary Figure 5 Activities change profile of metabolic pathways in comparison with berberine-exposed group (T1) vs. initial control group (C0).

Supplementary Figure 6 Volcano plots showing the trends of metabolites detected in the major metabolic pathways.

Supplementary Figure 7 Box plots for the major metabolic markers in *S. aureus* ATCC 25923 following berberine-exposure (T1) compared with non-exposed controls (C0, C1).

Supplementary Figure 8 Effect of BBR on pigment accumulation of *S. aureus* ATCC 25923.

Supplementary Figure 9 Expression profile of genes involved in staphyloxanthin biosynthesis in *S. aureus* ATCC 25923 responsive to berberine-exposure.

Supplementary Table 1 Parameters of OPLS-DA and RPT test.
