# Supplementary material for: A New Perspective on the Antimicrobial Mechanism of Berberine Hydrochloride Against Staphylococcus aureus Revealed by Untargeted Metabolomic Studies
Source: Front Microbiol. 2022 Jul 13;13:917414. doi: 10.3389/fmicb.2022.917414 (PMC9328669; doi:10.3389/fmicb.2022.917414)
Supplement: Supplementary Text 1 — Details for untargeted metabolomics analyses. [file Data_Sheet_1.pdf]

Text S1. Details for untargeted metabolomics analyses:

Metabolite derivatization for GC-MS. 80 µl of 15 mg/mL methoxylamine hydrochloride in pyridine was added to the freeze-dried samples. The resultant mixture were vortexed vigorously for 2 min and incubated at 37 °C for 90 min. 50 µL of BSTFA (with 1% TMCS) and 20 µL n-hexane was added into the mixture, which was vortexed vigorously for 2 min and then derivatized at 70 ℃ for 60 min. The samples were placed at ambient temperature for 30 min before GC-MS analysis.

GC-MS analysis. The derivative samples were analyzed by a Thermo Trace 1310/TSQ 9000 GC/MSD System. DB-5MS fused-silica capillary column (30 m × 0.25 mm × 0.25 µm, Agilent J & W Scientific, Folsom, CA, USA) was utilized to separate the derivatives. Helium (> 99.999%) was used as the carrier gas at a constant flow rate of 1 mL / min through the column. The injector temperature was maintained at 300 °C. Injection volume was 1 µL by splitess mode and maintained for 5 min. The programs were as followed: 60 ℃ initially for 0.5 min, increased to 125 ℃ at a rate of 8 ℃/min, to 210 ℃ at 4 ℃/min, to 270 ℃ at 10 ℃/min, to 305 ℃ at 20 ℃/min, and 305 ℃ was maintained for 3 min. The temperature of MS quadrupole, and ionsource (electronimpact) was set to 280, and 330 ℃, respectively. Mass data was acquired in a full-scan mode (m/z 50-500).

Metabolite re-extraction for LC-MS. 200 µL mixture of methanol and water (1/4,vol/vol) were added to each freeze-dried sample. Samples were vortexed for 30s, extracted by ultrasonic for 3 min in ice-water bath and then placed at -20 ℃ for 2h. After that, samples were centrifuged at 4 ℃ (13,000 rpm) for 10min. The supernatants (150 µL) from each tube were collected using crystal syringes, filtered through 0.22 µm microfilters and transferred to LC vials. The vials were stored at -80 ℃ until LC-MS analysis.

LC-MS analysis. A Nexera UPLC system (Shimadzu Corporation, Japan) coupled with Q-Exactive quadrupole-Orbitrap mass spectrometer equipped with heated electrospray ionization (ESI) source (Thermo Fisher Scientific, Waltham, MA, USA) was used in this study. An ACQUITY UPLC HSS T3 column (1.8 µm, 2.1×100 mm) were employed in both positive and negative modes. The binary gradient elution system consisted of (A) water (containing 0.1% formic acid, v/v) and (B) acetonitrile (containing 0.1 % formic acid, v/v) and separation was achieved using the following gradient: 0 min, 5% B; 2 min, 5% B; 4 min, 30% B; 8 min, 50% B;10 min, 80% B; 14 min, 100% B; 15 min, 100% B; 15.1 min, 5% and 16 min, 5% B. The flow rate was 0.35 mL/min and column temperature was 45 ℃. All the samples were kept at 4 ℃ during the analysis. The injection volume was 2 µL. The mass range was from m/z 100 to 1,200. The resolution was set at 70,000 for the full MS scans and 17,500 for HCDMS/MS scans. The Collision energy was set at 10, 20 and 40 eV. The mass spectrometer operated as follows: spray voltage, 3,500 V (+) and 3,000 V (−); sheath gas flow rate, 40 arbitrary units (+) and 35 arbitrary units (-); auxiliary gas flow rate, 10 arbitrary units (+) and 8 arbitrary units (-); capillary temperature, 320 ℃. The QCs were injected at regular intervals throughout the analytical run to provide a set of data from which repeatability can be assessed.
