# Supplementary figures and images for: A New Perspective on the Antimicrobial Mechanism of Berberine Hydrochloride Against Staphylococcus aureus Revealed by Untargeted Metabolomic Studies
Source: Front Microbiol. 2022 Jul 13;13:917414. doi: 10.3389/fmicb.2022.917414 (PMC9328669; doi:10.3389/fmicb.2022.917414)

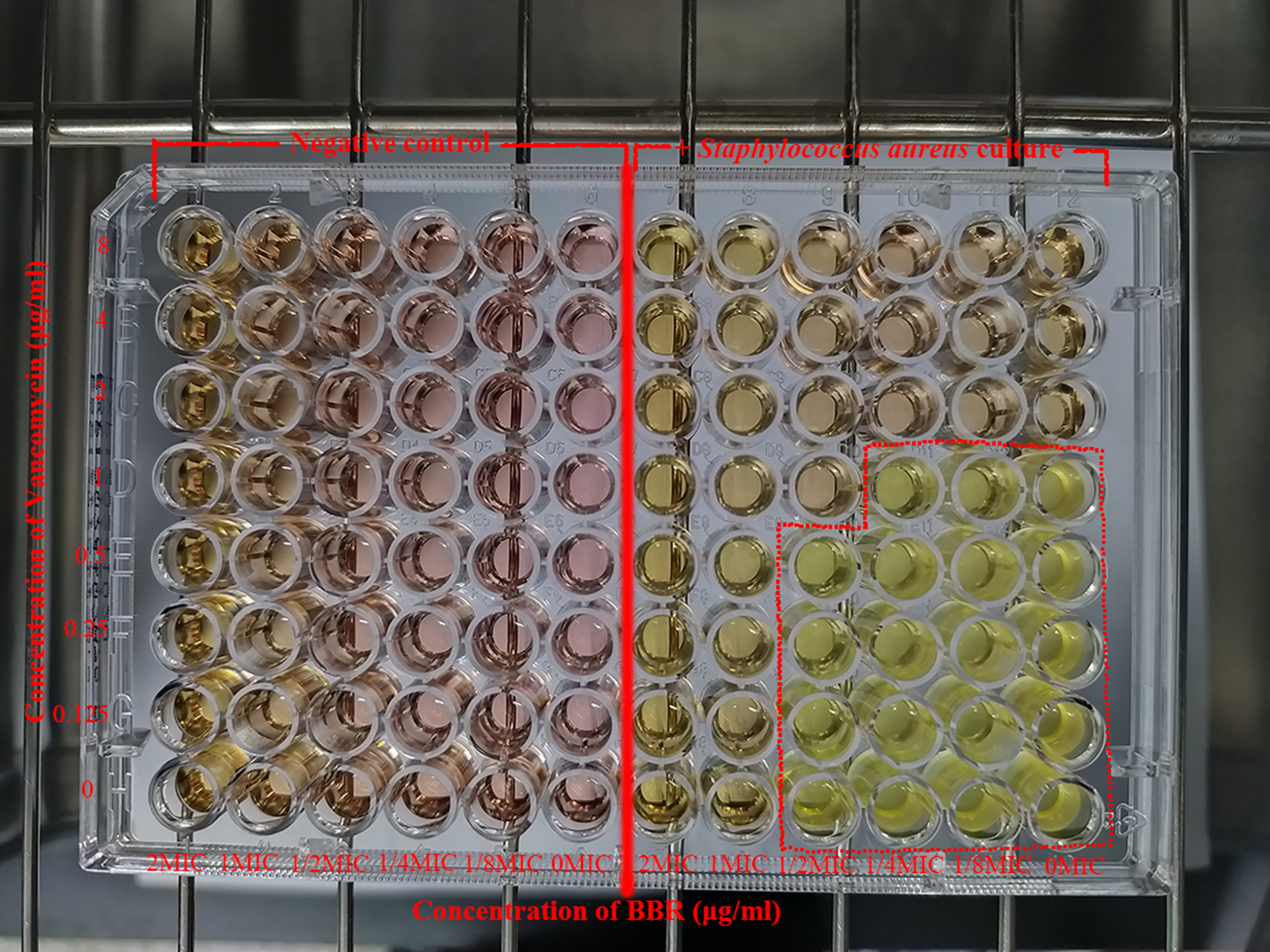

Supplement: Supplementary Figure 1 — MIC test of BBR combined with vancomycin against S. aureus ATCC 25923. [file Image_1.JPEG]

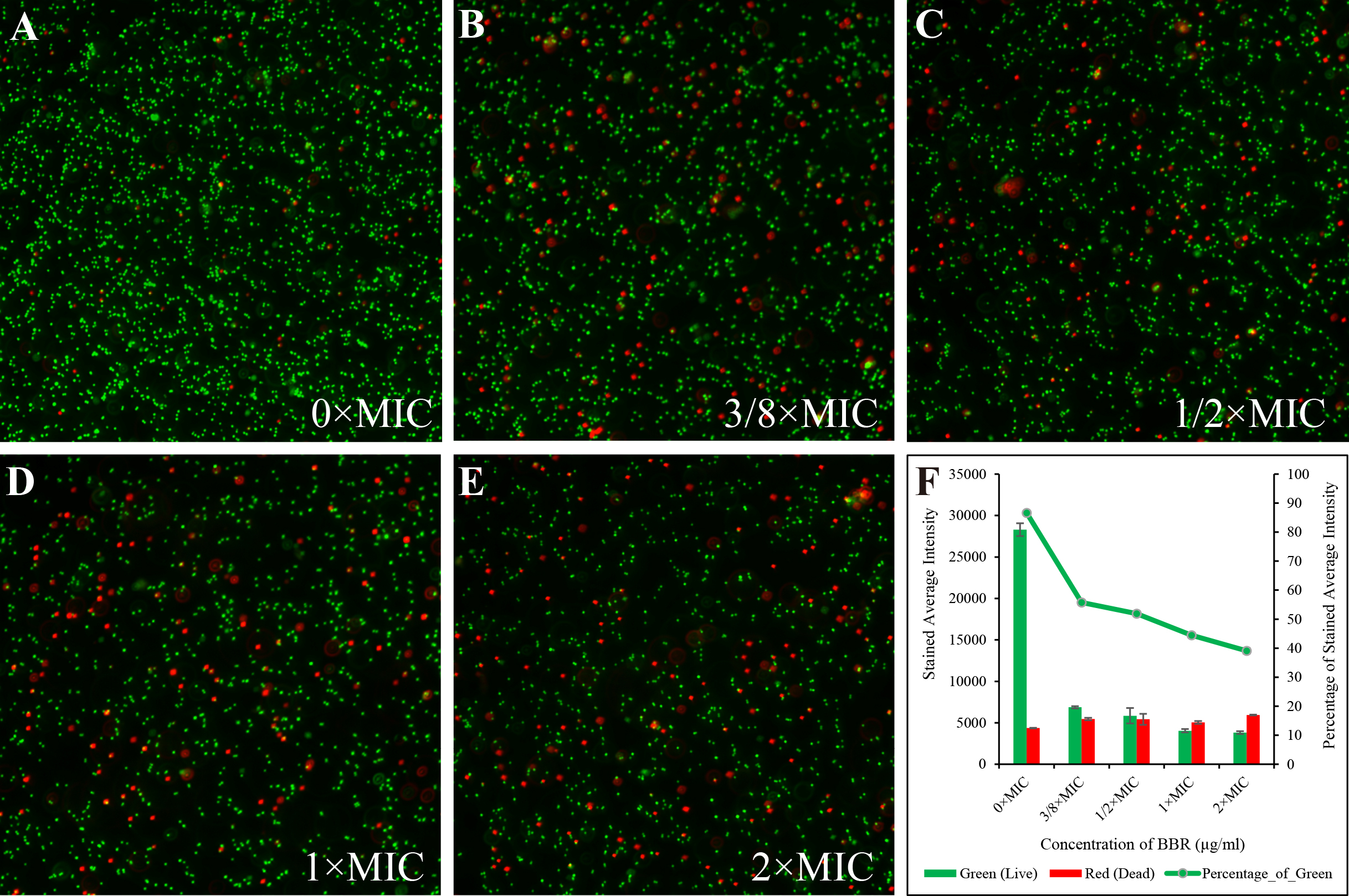

Supplement: Supplementary Figure 2 — The effect of BBR on survival of S. aureus ATCC 25923. [file Image_2.JPEG]

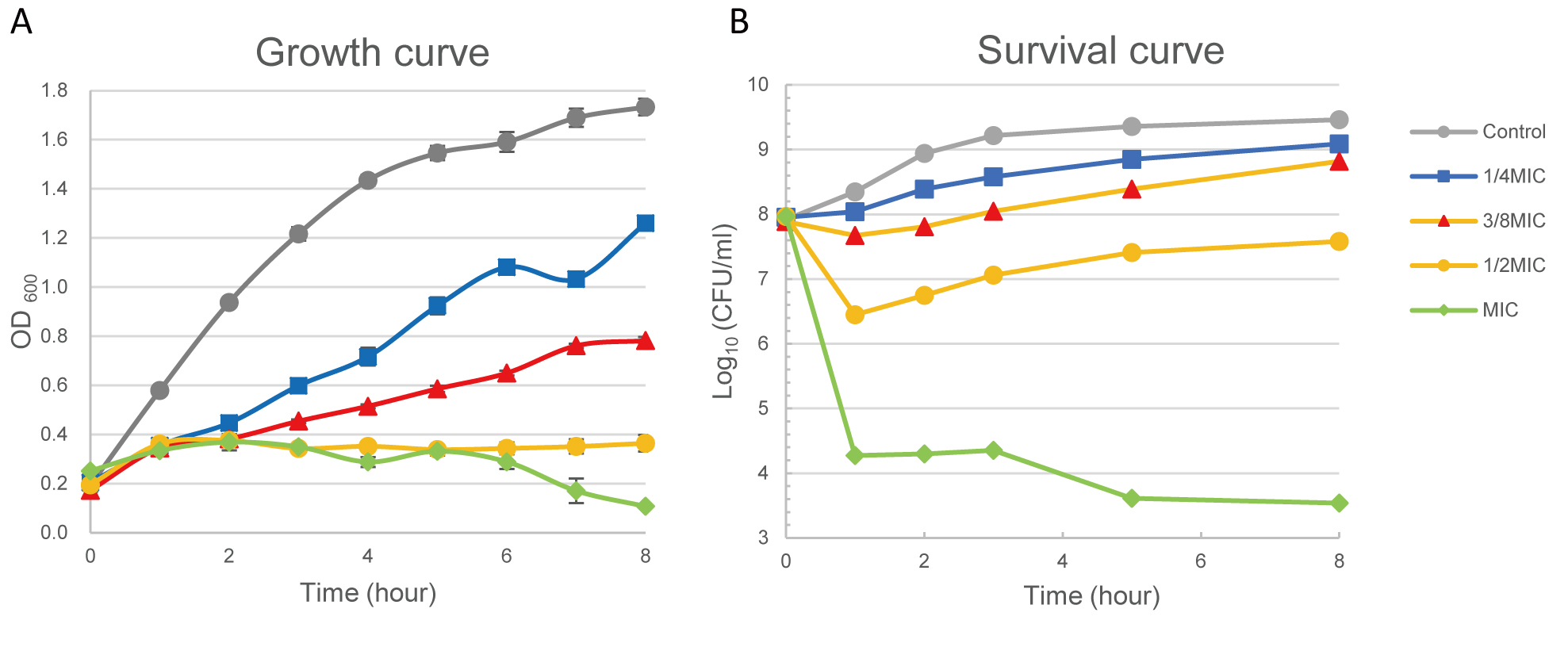

Supplement: Supplementary Figure 3 — Growth curves (A) and the results of time kill tests (B) for S. aureus ATCC 25923 in the presence (1/4MIC, 3/8MIC, 1/2MIC or MIC) or absence (control) of berberine. [file Image_3.JPEG]

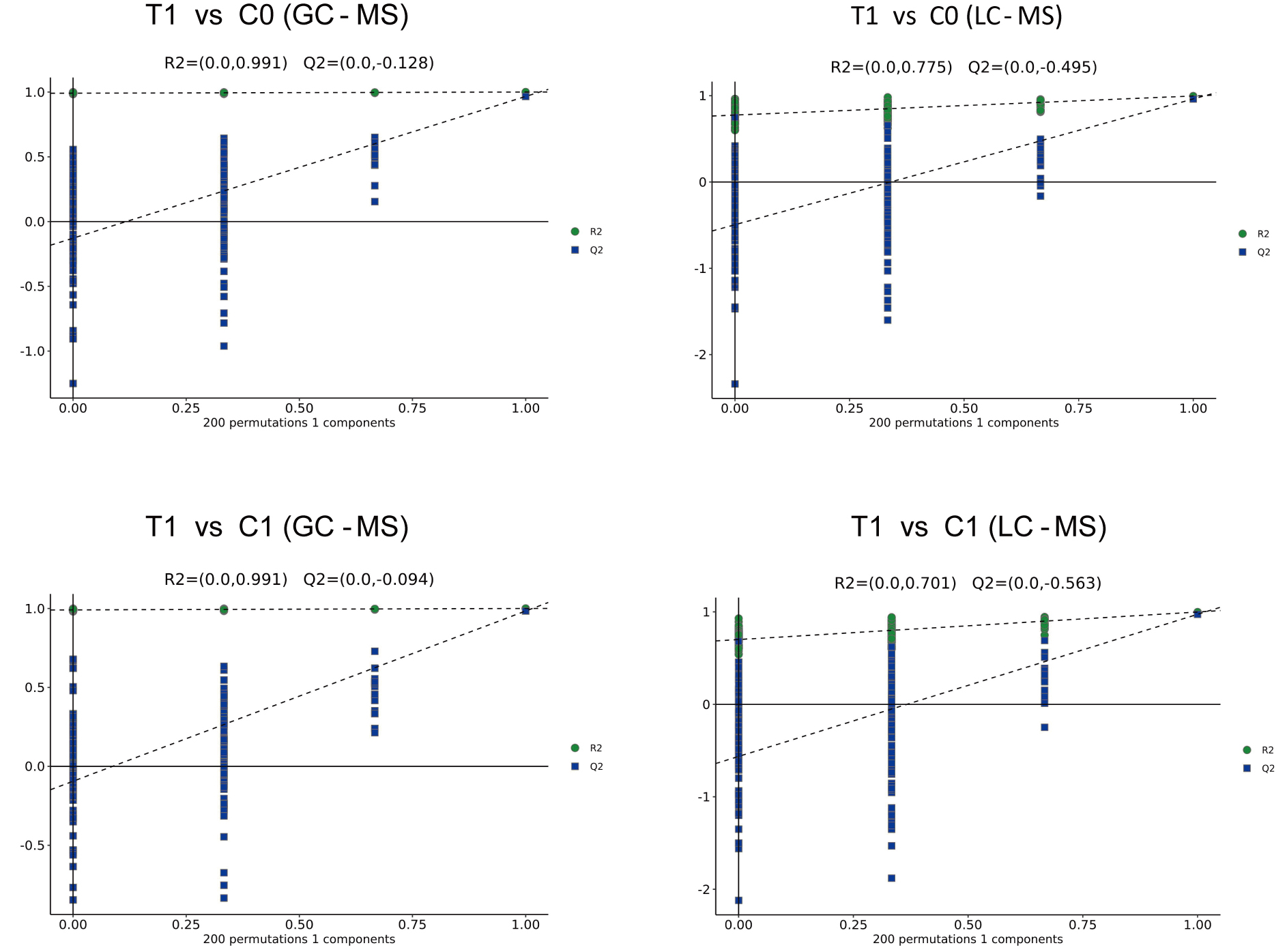

Supplement: Supplementary Figure 4 — Result of RPT. [file Image_4.JPEG]

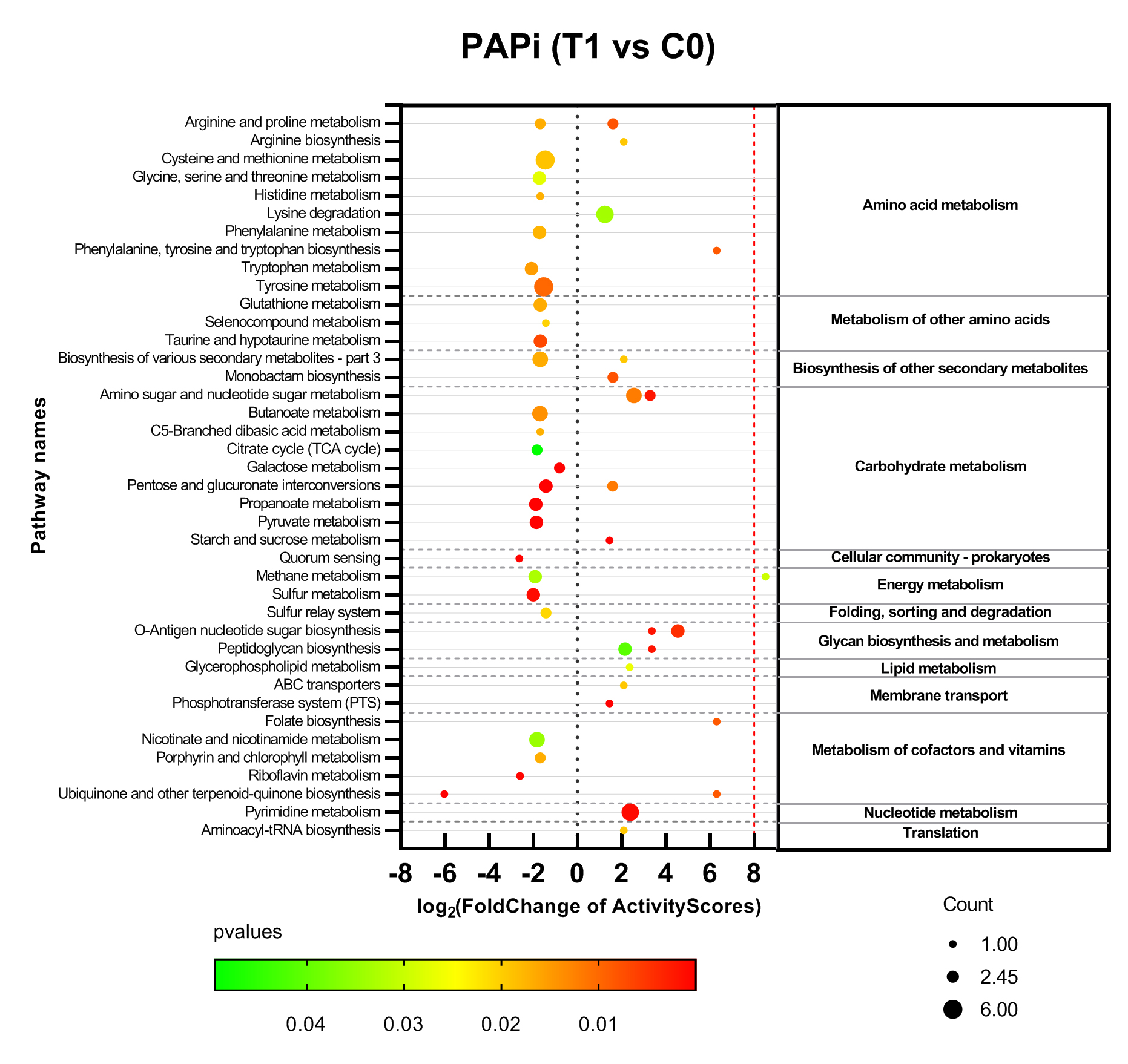

Supplement: Supplementary Figure 5 — Activities change profile of metabolic pathways in comparison with berberine-exposed group (T1) vs. initial control group (C0). [file Image_5.JPEG]

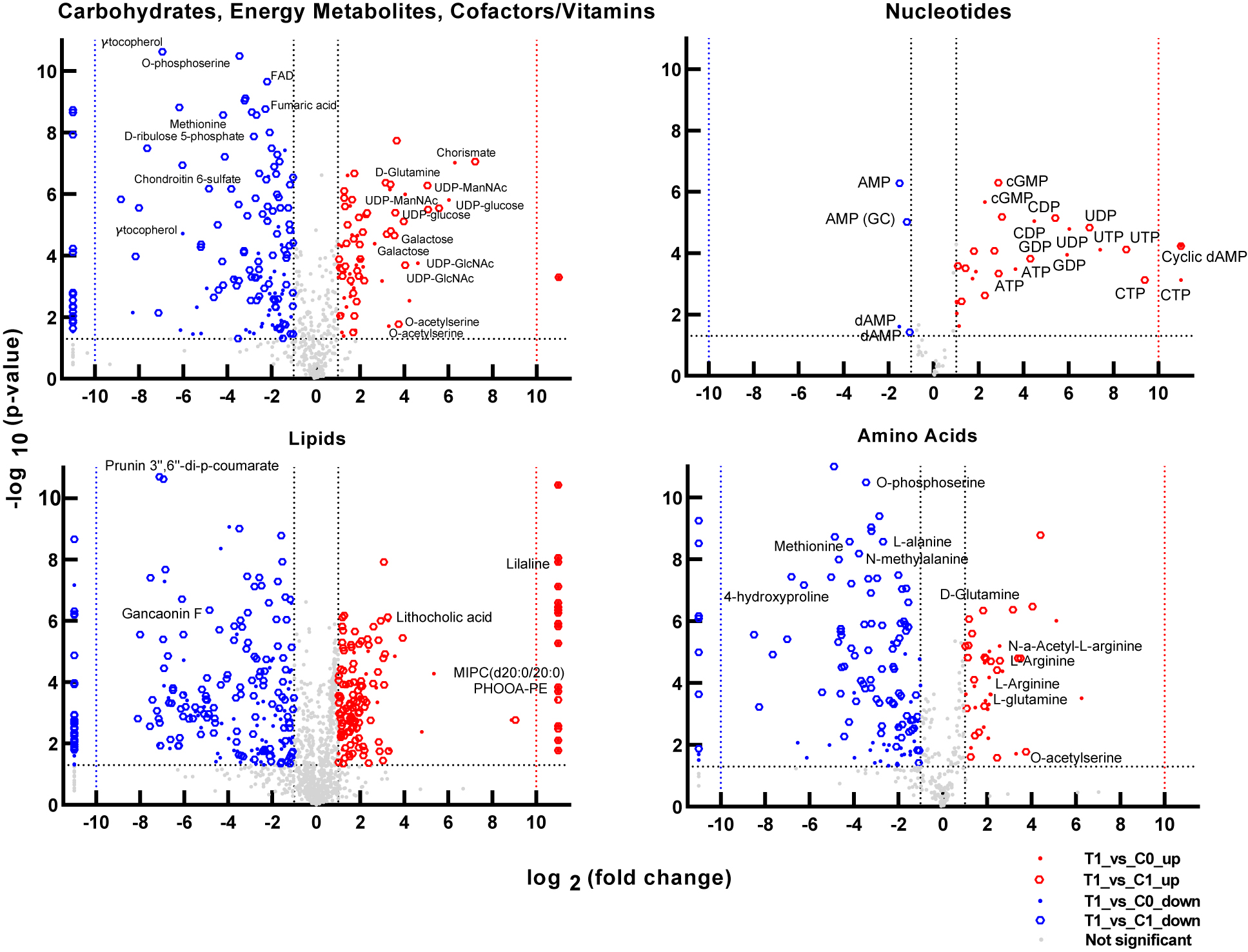

Supplement: Supplementary Figure 6 — Volcano plots showing the trends of metabolites detected in the major metabolic pathways. [file Image_6.JPEG]

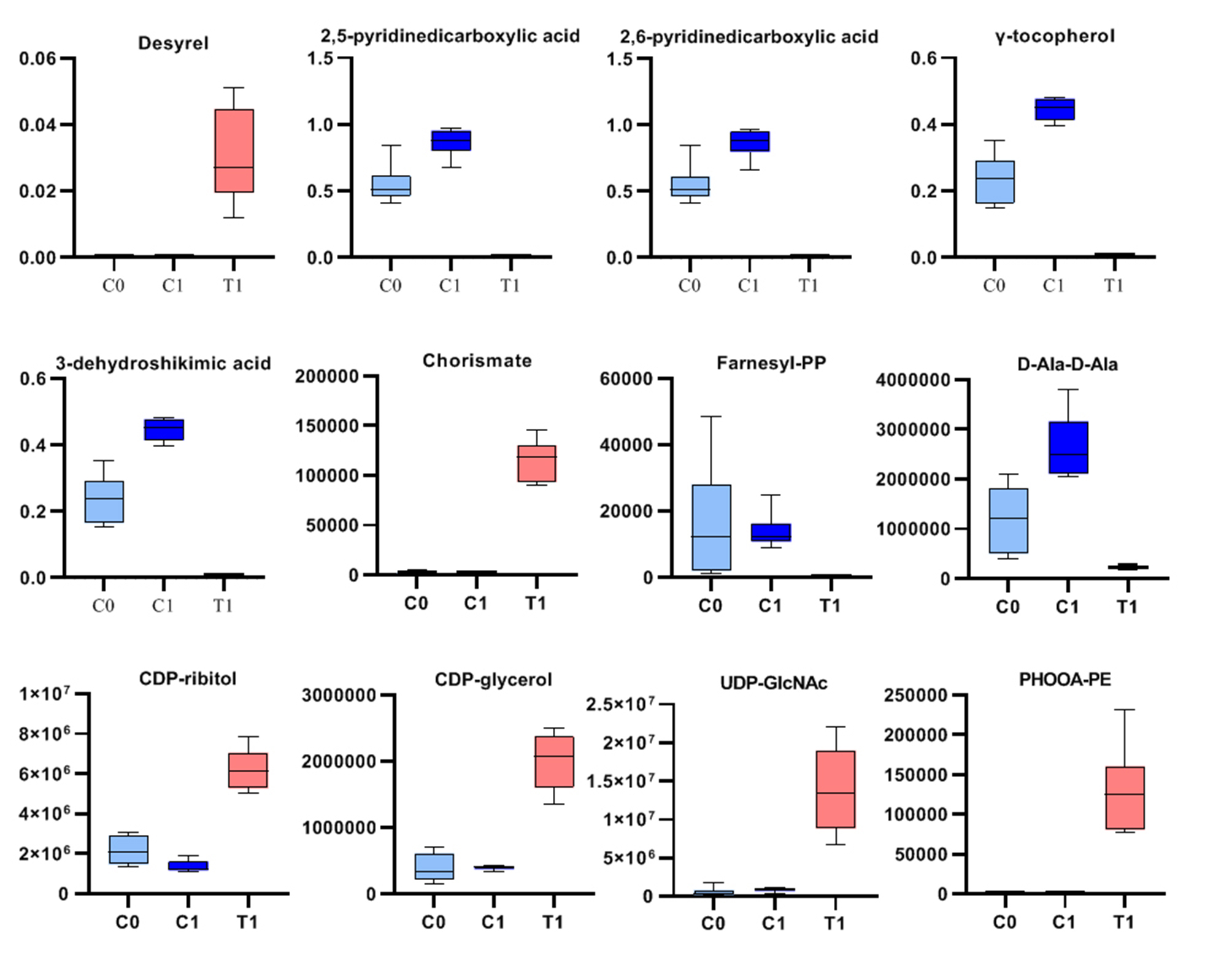

Supplement: Supplementary Figure 7 — Box plots for the major metabolic markers in S. aureus ATCC 25923 following berberine-exposure (T1) compared with non-exposed controls (C0, C1). [file Image_7.JPEG]

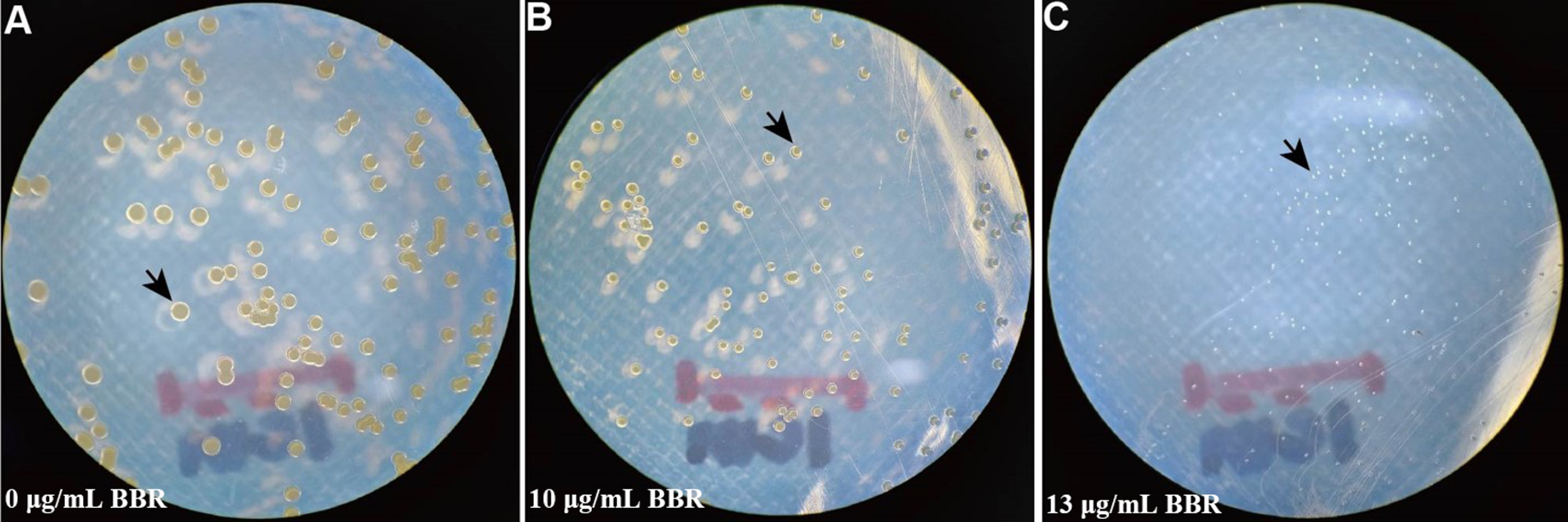

Supplement: Supplementary Figure 8 — Effect of BBR on pigment accumulation of S. aureus ATCC 25923. [file Image_8.JPEG]

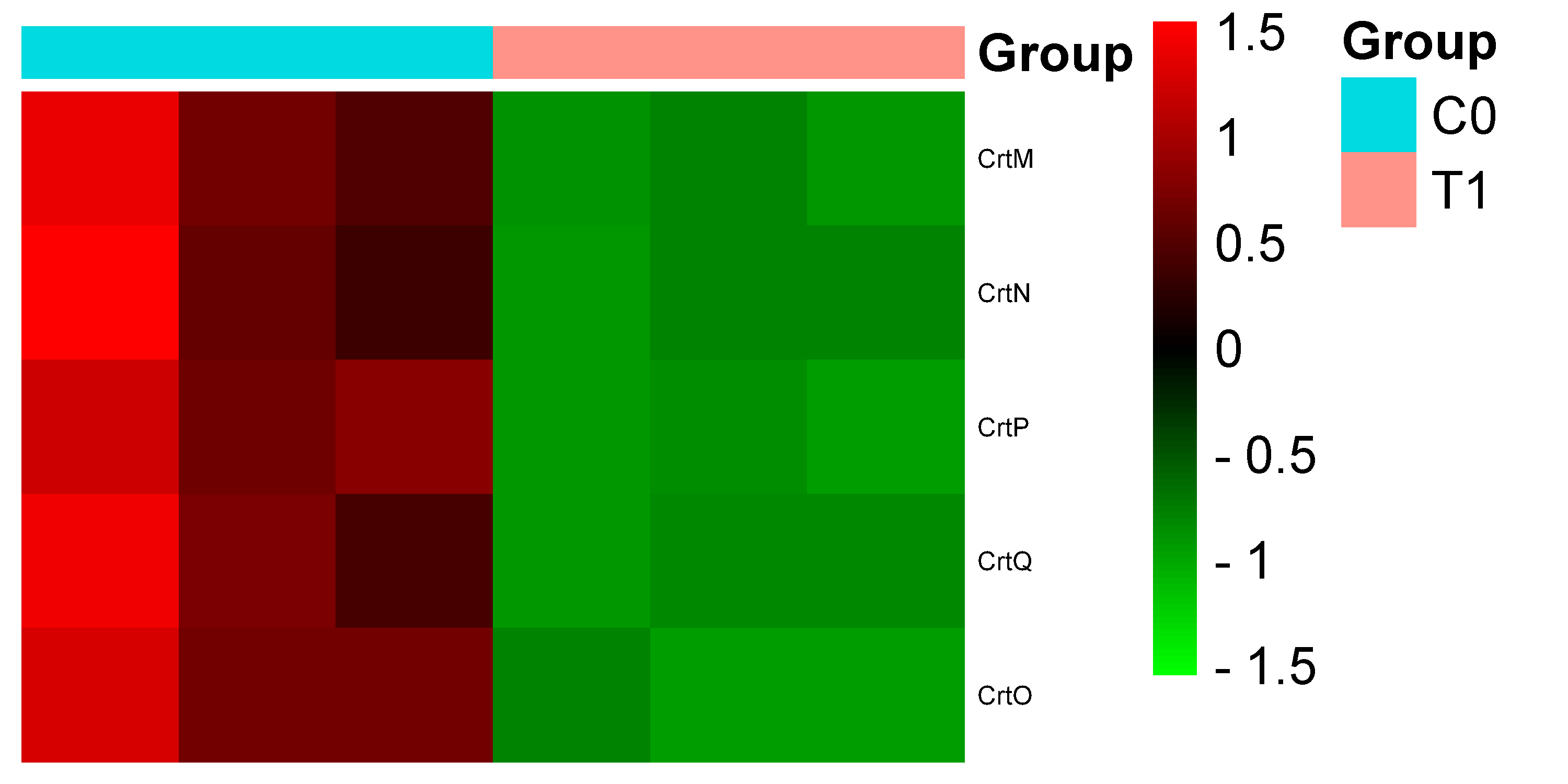

Supplement: Supplementary Figure 9 — Expression profile of genes involved in staphyloxanthin biosynthesis in S. aureus ATCC 25923 responsive to berberine-exposure. [file Image_9.JPEG]
