# Supplementary material for: A New Perspective on the Antimicrobial Mechanism of Berberine Hydrochloride Against Staphylococcus aureus Revealed by Untargeted Metabolomic Studies
Source: Front Microbiol. 2022 Jul 13;13:917414. doi: 10.3389/fmicb.2022.917414 (PMC9328669; doi:10.3389/fmicb.2022.917414)
Supplement: Supplementary Table 1 — Parameters of OPLS-DA and RPT test. [file Table_1.DOCX]

**Supplementary** **Table 1.** Parameters of OPLS-DA and RPT tests

| Database | Group | PRE | ORT | N | R2X(cum) | R2Y(cum) | Q2(cum) | R2(RPT) | Q2(RPT) |
| --- | --- | --- | --- | --- | --- | --- | --- | --- | --- |
| LC-MS | T1 vs C0 | 1 | 2 | 12 | 0.862 | 0.996 | 0.962 | 0.775 | -0.495 |
| LC-MS | T1 vs C1 | 1 | 2 | 12 | 0.923 | 0.999 | 0.974 | 0.701 | -0.563 |
| GC-MS | T1 vs C0 | 1 | 2 | 12 | 0.551 | 1 | 0.966 | 0.991 | -0.128 |
| GC-MS | T1 vs C1 | 1 | 2 | 12 | 0.679 | 1 | 0.984 | 0.991 | -0.094 |

When model parameters of both R2Y (cum) and Q2 (cum) are greater than 0.5 and close to 1 means the prediction ability of the OPLS-DA model is excellent. When using the RPT test, it is generally required that the Q2 (RPT) is less than zero
